# Acute cerebral arterial embolism following pemetrexed and carboplatin treatment in non-small-cell lung cancer: A case report

**DOI:** 10.3892/mco.2013.147

**Published:** 2013-07-15

**Authors:** MASAYUKI TAKEDA, TAKEHIKO KOBAYASHI, SATOSHI MARUMO, YOSHIHIKO KOSHIMO, TAKASHI TERANISHI, YUICHI HIGAMI, MOTOKAZU KATO

**Affiliations:** 1 Department of Medical Oncology, Kishiwada City Hospital, Kishiwada, Osaka 596-8501, Japan; 2 Department of Respirology, Kishiwada City Hospital, Kishiwada, Osaka 596-8501, Japan

**Keywords:** cerebral arterial embolism, chemotherapy, non-small-cell lung cancer

## Abstract

Thromboembolism is a known vascular toxicity associated with tumor chemotherapy. The combination of pemetrexed and carboplatin has exhibited significant antitumor activity, with mild manageable toxicity in patients with advanced non-small-cell lung cancer (NSCLC), whereas cerebral arterial embolism has not been recognized as a side effect associated with this regimen. This is the case report of an unusual case of NSCLC, in which the patient suffered a left middle cerebral arterial embolism following chemotherapy. A 62-year-old non-smoking woman, diagnosed with stage IV lung adenocarcinoma, was administered pemetrexed and carboplatin as second-line therapy. On the day of the completion of the first regime cycle, the patient was readmitted to the emergency department with complaints of sudden-onset right hemiplegia and agitation. Brain magnetic resonance imaging and magnetic resonance angiography revealed an occlusion of the left middle cerebral artery (MCA) and no further chemotherapy was administered due to the deterioration in the performance status of the patient associated with right hemiplegia. Pemetrexed plus carboplatin is routinely used for the treatment of advanced NSCLC. The present case highlights the potential risk for development of embolism following pemetrexed-based chemotherapy. Further investigations are required to elucidate the mechanism through which these drugs may eventually cause neurovascular adverse events. Clinicians should be aware of the potential risk for development of cerebral arterial embolism following pemetrexed-based chemotherapy.

## Introduction

Thromboembolism is a known vascular toxicity associated with tumor chemotherapy. The combination of pemetrexed and carboplatin has exhibited significant antitumor activity, with mild manageable toxicity in patients with advanced non-small-cell lung cancer (NSCLC) (1), whereas cerebral arterial embolism has not been recognized as a side effect associated with this regimen. This is the case report of an unusual case of NSCLC, in which the patient suffered a left middle cerebral arterial embolism following administration of pemetrexed and carboplatin.

## Case report

A 62-year-old non-smoking woman was diagnosed with stage IV adenocarcinoma of the lung in October, 2011. The patient had no previous medical history of arrhythmia, ischemic heart disease, diabetes mellitus or stroke and did not receive any daily medications. Mutation analysis of lung cancer specimens obtained prior to the administration of first-line chemotherapy revealed the presence of an L858R point mutation of the epidermal growth factor receptor gene. The patient was administered gefitinib (250 mg daily) as a first-line therapy; however, progressive multiple brain metastases were detected after 4 months of therapy. Following whole-brain radiation therapy (WBRT) at a total dose of 30 Gy, the patient resumed gefitinib treatment. Five months after WBRT, positron emission tomographic and computed tomographic (CT) imaging revealed disease progression in the liver and bone. An echocardiogram prior to administration of second-line chemotherapy revealed no thrombus in the heart, no atrioventricular septal defects, no patent foramen ovale and no valvular vegetations. The combination of pemetrexed and carboplatin was then administered as second-line treatment.

**Figure 1:**
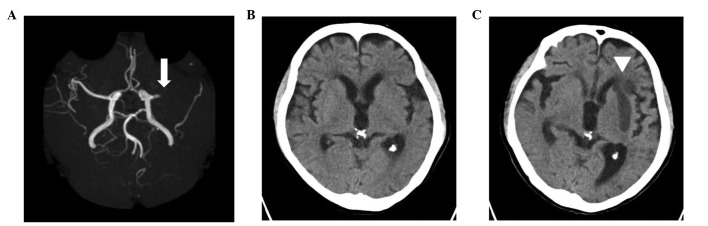
(A) Brain magnetic resonance angiography showing an occlusion of the left middle cerebral artery (MCA) (arrow) on the day of stroke. (B) Brain computed tomography (CT) revealed no new abnormalities on the day of the stroke. (C) CT scan 6 days after the stroke revealed the presence of an infarction in the supply area of the MCA (arrowhead).

On the day of the completion of the first regime cycle, the patient was readmitted to the emergency department with complaints of sudden-onset right hemiplegia and agitation. Laboratory testing revealed grade 1 anemia (hemoglobin, 11.3 g/dl), grade 1 thrombocytopenia (116,000/μl), a slight elevation of the prothrombin time/international normalized ratio to 1.18 (normal range, 0.90–1.10), a substantial increase in D-dimer levels to 19.2 μg/ml (normal range, 0–0.9 μg/ml) and fibrin degradation product (FDP) levels of 47.8 μg/ml (normal range, 0.1–4.99 μg/ml). A non-contrast brain CT revealed no new abnormalities (Fig. 1B); however, brain magnetic resonance imaging and magnetic resonance angiography revealed an occlusion of the left middle cerebral artery (MCA) (Fig. 1A). Following consultation with a neurologist and considering the substantial risk of cerebral hemorrhage caused by thrombolytic agents, the patient was not administered thrombolytic therapy. Six days after the stroke, a cranial CT scan revealed the presence of an infarction in the supply area of the MCA (Fig. 1C). No further chemotherapy was administered due to the deterioration in the performance status of the patient associated with right hemiplegia and the patient succumbed to progressive cancer 3 months after the MCA embolism.

Written informed consent was obtained from the patient for publication of this case report and accompanying images.

## Discussion

In the present case, the patient suffered an MCA embolism on the day of completion of the first cycle of chemotherapy with pemetrexed and carboplatin. The mechanisms underlying the cerebrovascular event secondary to antineoplastic agent administration are likely multifactorial, including tumor microemboli, radiation-induced vasculopathy and thromboembolism.

The suggested mechanism of systemic tumor embolization from lung cancer is invasion of the pulmonary veins, with or without invasion of the left atrium (2). Rarely, a tumor may invade the venous circulation and spread to the left heart through a patent foramen ovale, leading to systemic tumor embolization. The risk of tumor embolization in the present case appeared to be low, since a chest CT scan had revealed no evidence of pulmonary vein or left atrial invasion by lung cancer.

Cumulative radiation-induced vascular damage in terms of endothelial lesions may be another factor. An occlusive vasculopathy with accelerated atherosclerosis may affect cranial vessels within the irradiated field. Pathological studies demonstrated that radiotherapy produces a sequence of arterial changes characterized by initial endothelial cell damage, thickening of the intimal layer caused by smooth muscle cell proliferation, cell degeneration and hyaline transformation. Intracranial occlusive vasculopathies associated with whole-brain irradiation, gamma knife or other focused cranial radiation were also reported (3,4).

Another possible explanation in the present case is that thromboembolism may have accounted for the cerebrovascular event. Platelet activation, alteration of the clotting cascade, including hyperfibrinolysis, and disturbance of the prostacyclin-thromboxane homeostasis, contribute towards an increased risk of thrombosis by 4- to 6-fold in cancer patients. compared to that in the general population (5). However, cerebral arterial embolism has not been recognized as a side effect associated with this regimen. Increased D-dimer and FDP levels may indicate the presence of an abnormal clot. However, it was difficult to determine the cause of the cerebral arterial embolism in the present case, since an autopsy was not performed.

In conclusion, pemetrexed plus carboplatin is routinely used for the treatment of advanced NSCLC. The present case highlights the potential risk for the development of embolism following pemetrexed-based chemotherapy. Future studies should be conducted to elucidate the mechanisms through which these drugs may eventually cause neurovascular adverse events.

## References

[1] Gronberg BH, Bremnes RM, Flotten O (2009). Phase III study by the Norwegian lung cancer study group: pemetrexed plus carboplatin compared with gemcitabine plus carboplatin as first-line chemotherapy in advanced non-small-cell lung cancer. J Clin Oncol.

[2] Al-Thenayan E, Maghfoor I (2004). Complications of malignancy: case 1. Systemic tumor embolism from lung cancer at presentation. J Clin Oncol.

[3] Bernstein M, Lumley M, Davidson G, Laperriere N, Leung P (1993). Intracranial arterial occlusion associated with high-activity iodine-125 brachytherapy for glioblastoma. J Neurooncol.

[4] Omura M, Aida N, Sekido K, Kakehi M, Matsubara S (1997). Large intracranial vessel occlusive vasculopathy after radiation therapy in children: clinical features and usefulness of magnetic resonance imaging. Int J Radiat Oncol Biol Phys.

[5] Blom JW, Doggen CJ, Osanto S, Rosendaal FR (2005). Malignancies, prothrombotic mutations, and the risk of venous thrombosis. JAMA.

